# Bi-functional particles for real-time phagosome acidification and proteolysis multiplex assay in macrophages

**DOI:** 10.3389/fimmu.2023.1204223

**Published:** 2023-08-10

**Authors:** Alba Méndez-Alejandre, Benjamin Bernard Armando Raymond, Matthias Trost, José Luis Marín-Rubio

**Affiliations:** ^1^ Laboratory for Biological Mass Spectrometry, Biosciences Institute, Newcastle University, Newcastle-upon-Tyne, United Kingdom; ^2^ Biology Department, Autonomous University of Madrid, Madrid, Spain

**Keywords:** phagosome, proteolysis, acidification, macrophages, bacteria

## Abstract

Phagosome acidification and proteolysis are essential processes in the immune response to contain and eliminate pathogens. In recent years, there has been an increased desire for a rapid and accurate method of assessing these processes in real-time. Here, we outline the development of a multiplexed assay that allows simultaneous monitoring of phagosome acidification and proteolysis in the same sample using silica beads conjugated to pHrodo and DQ BSA. We describe in detail how to prepare the bi-functional particles and show proof of concept using differentially activated macrophages. This multiplexed spectrophotometric assay allows rapid and accurate assessment of phagosome acidification and proteolysis in real-time and could provide valuable information for understanding the immune response to pathogen invasion.

## Introduction

Phagocytosis is an essential process for the innate immune system, as it helps to protect the body from pathogens and other harmful substances. Additionally, this process is essential for tissue repair, to regulate the inflammatory response and to present antigens to other immune cells. In this process, cells engulf and absorb foreign particles larger than 0.5 μm in diameter, such as microorganisms, foreign particles, or cellular debris (1). While many types of cells are able to phagocytose, this is largely performed by professional phagocytes such as macrophages, neutrophils, monocytes, dendritic cells, and osteoclasts. Macrophages can recognise and engulf foreign particles, as well as release cytokines that help to activate other cells in the immune response (2). After phagocytic uptake, the newly formed phagosome undergoes a series of changes in protein and membrane composition, termed phagosome maturation (3–6). The process begins when a cell binds to a particle and forms a phagocytic cup around it, then uses its pseudopods to engulf the particle, allowing it to be pulled into the cell. In the early stages, the phagosome membrane fuses with endocytic vesicle membranes that contain important factors such as the v-ATPases, which pump protons (H^+^) from the cytosol and acidify the lumen of the phagosome (reaching pH~5), and the major histocompatibility complex (MHC) proteins, which are essential for antigen presentation (5, 7). The phagosome also increases in size, allowing it to accommodate more material and absorb more substances (6). Finally, the phagosome fuses with the lysosome forming the phagolysosome, which delivers hydrolytic enzymes such as proteases, nucleases, glycosidases, lipases, and reactive oxygen species (ROS) to kill and digest the pathogens (3, 8). In this stage, proteases can only be activated under acidic conditions to digest the pathogen and process the antigen for its presentation (6). Then, they can also activate the adaptive immune system and help the body effectively fight off foreign invaders. It is therefore an essential process for the survival and protection of the cell.

The acidification and proteolysis of phagosomes in phagocytic cells are key processes that have important implications for cellular homeostasis. When phagocytic cells are unable to properly acidify and proteolyse phagosomes, it can lead to alterations in the immune response to infections and pathogens, which can have adverse clinical consequences (9–11). In order to better understand these processes, a variety of techniques have been implemented to monitor single processes, including immunoblotting, quantitative PCR, and flow cytometry to measure changes in the levels of proteolytic enzymes, changes in the mRNA expression of proteolytic enzymes or changes in phagosome formation and determine the acidification state of the phagosome (12–14). Additionally, other techniques have been used to visualise the uptake of fluorescently labelled particles into phagosomes and track changes in their acidification and maturation. These assays rely on pH-sensitive fluorescent dyes, particles bound to *E. coli*, bioparticles labelled with pH-sensitive and pH-insensitive fluorochromes (12, 15–17). However, none of these methods allows for the investigation of both acidification and proteolysis in parallel.

To address this need, we report here a detailed method to prepare bi-functional particles for the simultaneous quantification of pH and proteolytic activity inside phagosomes during maturation using a spectrophotometer. For this, we coated silica beads with pHrodo and DQ BSA to measure the acidity and hydrolysis activity in phagocytic cells.

## Materials and equipment

### Materials and equipment

· 50 mg/mL (1 – 3x10E9 beads/mL), 3.0 µm carboxylate-modified silica particles (Kisker Biotech).

· 96-well black clear bottom polystyrene microplates (Corning Life Sciences).

· Spectrophotometer, SpectraMax Gemini EM (Molecular Devices).

· FACSymphony A5 flow cytometer (Becton-Dickinson).

· LSM 800 super-resolution microscopy with Airyscan (Zeiss).

### Primary macrophages and macrophage cell line

· Bone marrow differentiated macrophages (BMDMs) (18) from 10 to 12-week-old C57BL/6NTac mice (UKRI-MRC Harwell). Newcastle University ethical committee approved animal work and manipulation was performed under UK Home Office project licence.

· RAW 264.7 (ATCC, TIB-71) and THP-1 (ATCC, TIB-202) cells. ATCC routinely performs cell line authentication, using short tandem repeat profiling as a procedure. Cell experimentation was always performed within a period not exceeding 6 months after resuscitation in mycoplasma-free culture conditions.

### Bacterial strains and culture conditions

· *Salmonella enterica* serovar Typhimurium SL1344 strain was kindly provided by Dirk Bumann (Biozentrum Basel) and grown in Luria-Bertani (LB) broth at 37°C with constant rotation.

· *Staphylococcus aureus* RN6390 strain was kindly provided by Tracy Palmer (Newcastle University) and grown in Tryptone Soy Broth (TSB) at 37°C with constant rotation.

All bacteria were used at mid-exponential phase, washed in ice cold PBS twice and, subsequently, re-suspended in 4% PFA in PBS for 15 min.

### Reagents and buffers

· Dubecco’s modified eagle medium (DMEM).

· Roswell Park Memorial Institute (RPMI).

· Iscove’s Modified Dulbecco’s Media (IMDM).

· 100 U/ml penicillin-streptomycin.

· Foetal bovine serum (FBS).

· L-Glutamine at a final concentration of 2 mM.

· Interferon gamma (IFN-γ) (PeproTech), at a final concentration of 20 μg/mL.

· Interleukin 4 (IL-4) (PeproTech), at a final concentration of 20 μg/mL.

· Binding buffer: 1 mM CaCl2, 2.7 mM KCl, 0.5 mM MgCl2, 5 mM dextrose, 10 mM HEPES and 10% FBS in PBS pH 7.2.

· Sodium borate buffer: 100 mM boric acid in purified water, pH 8.0.

· Glycine buffer: 250 mM glycine in PBS pH 7.2.

· Cyanamide buffer: 25 mg/mL of cyanamide in purified water, made fresh.

· Sodium azide 1% in purified water, made fresh.

· 5 mg/mL Alexa Fluor 405 (AF405) carboxylic acid, succinimidyl ester (Invitrogen) in DMSO.

· 2 mg/mL, DQ red BSA (Invitrogen) in sodium borate buffer.

· 5 mg/mL pHrodo green, succinimidyl ester (Invitrogen) in DMSO.

· Bafilomycin A1 (Sigma-Aldrich), at a final concentration of 100 µM.

· 100 µg/mL LPS (Invitrogen).

· 100 µg/mL Pam_3_CSK_4_ (Invitrogen).

· Leupeptin (Sigma-Aldrich), at a final concentration of 100 µM.

## Methods

### Preparation of bi-functional particles and fixed bacteria

1. Take 100 µL of 3.0 µm carboxylate-modified silica beads or 2.5 x 10^9^ PFA-fixed bacteria.

2. Wash the beads three times with 1 mL PBS at 2,000 x g for 1 min using a benchtop centrifuge.

3. Incubate the beads with 500 µL of the heterobifunctional crosslinker cyanamide (cyanamide buffer) for 15 min under constant agitation at 900 rpm at room temperature (**Figure 1.1**).

**Figure 1:**
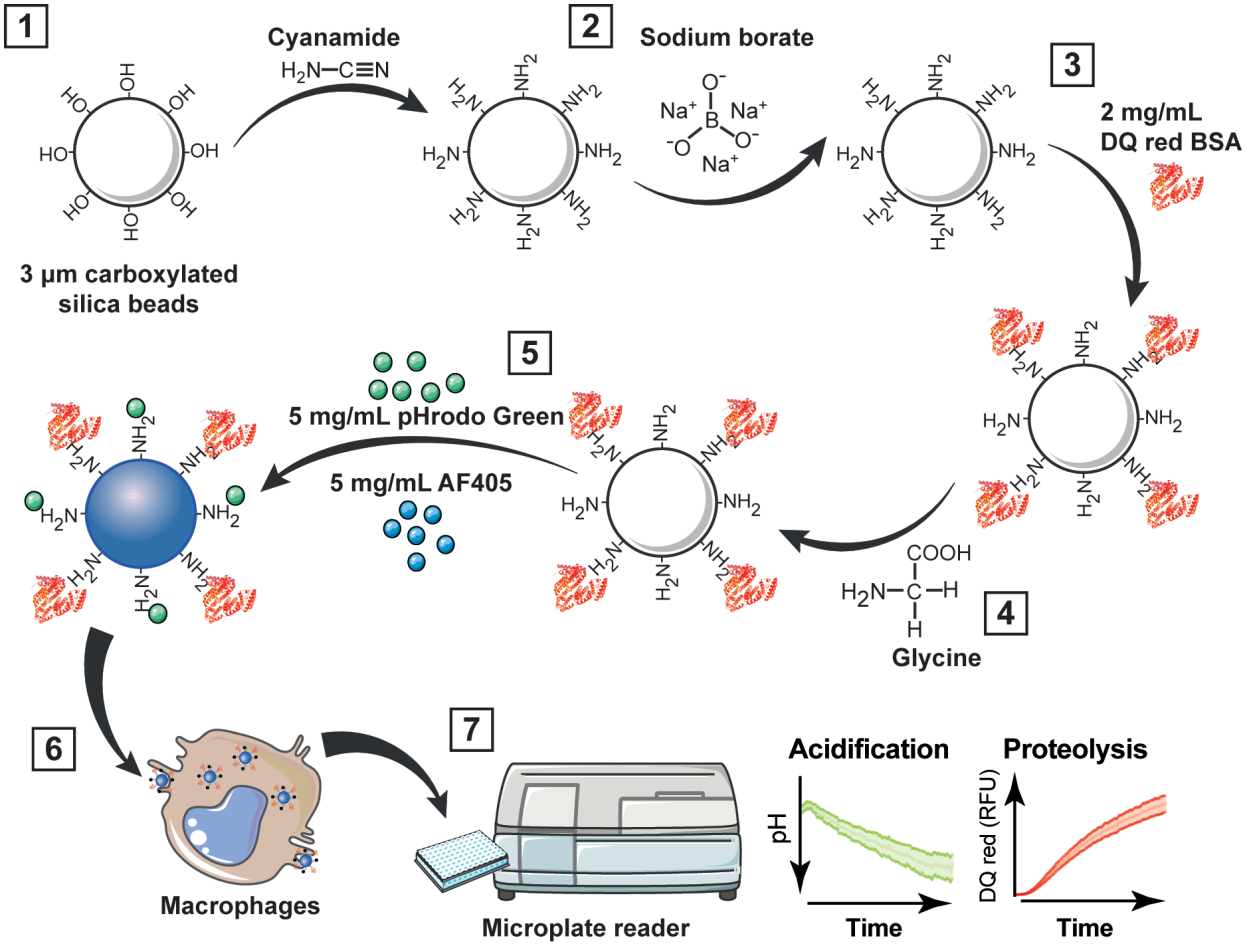
Workflow for multiplexing analysis of phagosome acidification and proteolysis. (1) Carboxyl functionalized silica nanoparticles or micro beads are wash with the heterobifunctional crosslinker cyanamide; (2) excess amines are removed by washing with sodium borate; then, (3) beads are incubated overnight with 2 mg/mL DQ red BSA in sodium borate buffer. (4) After the incubation, the beads are washed with glycine to quench unreacted cyanamide; and (5) incubated with 5 mg/mL pHrodo green and 5 mg/mL AF405 in sodium borate buffer for 1 h. (6) Bi-functional particles are added to macrophages, and (7) acidification and proteolysis are measured in real time simultaneously using a fluorescence microplate reader. RFU, relative fluorescence units.

4. Wash twice with 1 mL of sodium borate buffer at 2,000 x g for 3 min to remove soluble amine groups (**Figure 1.2**).

Note. The beads can be also coated with 1 mL of 100 µg/mL LPS or 100 µg/mL Pam3CSK4 in sodium borate buffer for 18 h at room temperature. Then washed twice with 1 mL of sodium borate buffer at 2,000 x g for 3 min to remove the unbound LPS or Pam3CSK4.

5. Incubate the beads with 500 µL of 2 mg/mL DQ red BSA in a sodium borate buffer for 18 h under constant agitation (900 rpm) at room temperature in the dark (**Figure 1.3**).

6. After the incubation, wash the beads twice with a glycine buffer at 2,000 x g for 2 min to quench unreacted cyanamide (**Figure 1.4**)

7. Wash the beads twice with a sodium borate buffer at 2,000 x g for 3 min to remove soluble amine groups.

8. Incubate the beads with 1 mL of 5 mg/mL pHrodo green and 5 mg/mL AF405 for 1 h in sodium borate buffer under constant agitation (900 rpm) at room temperature in the dark (**Figure 1.5**).

Note. N-hydroxysuccinimide (NHS) esters are commonly used for efficient conjugation of primary amines and typically activated at pH 7–9, which is the optimal range for their reactivity. When an active ester reacts with a primary amine, it forms a stable and irreversible amide bond. This amide bond formation is a crucial step in various bioconjugation reactions. However, they do not directly react with carbonyl groups. Once the primary amine is introduced onto the carbonyl-containing molecule, the NHS ester can be added, and the reaction proceeds to form the amide bond between the primary amine and the NHS ester (19, 20).

9. Wash the beads twice in PBS at 2,000 x g for 3 min.

10. Resuspend in 500 µL PBS with 1% sodium azide and stored in low-binding tubes at 4°C within a period of not more than one month.

### Fluorogenic kinetic assay in live cells

1. RAW 264.7, BMDMs or THP-1 cells were seeded on 96-well black clear bottom polystyrene microplates at 100,000 viable cells per well (**Figure 1.6**).

2. Treat cells with 20 μg/ml interferon gamma (IFN-γ) or 20 μg/ml interleukin 4 (IL-4) for 24 h and 48 h before the assay.

3. Remove medium and wash cells twice with a warm Binding buffer.

4. Add 100 μM bafilomycin A1 for 1 h before the assay as a v-ATPase inhibitor of vesicle acidification (negative control for acidification), which is one aspect of phagosome maturation, but it does not completely block the overall process of phagosome maturation. Moreover, 100 μM leupeptin for 1 h before the assay as a protease inhibitor can be used as a negative control for proteolysis. Maintain the treatment for the rest of the experiment.

5. Add bi-functional particles at a dilution of 1:200 (2 – 6 x 10E5 beads) in the Binding buffer and incubate for 30 min at 37°C in the incubator to allow the particles to attach to the macrophages.

6. Wash the cells twice with a Binding buffer.

Note. To validate the uptake of these bi-functional particles inside the cells, RAW 264.7 cells were imaged on a Zeiss LSM800 at 40X (oil immersion) or 63X (oil immersion) using Zen software with the following lasers set to 1% power: 405 nm, 488 nm, and 561 nm. The laser power was set to 1% of the maximum output (equivalent to 1 mW) to achieve optimal signal-to-noise ratio and minimise photobleaching effects. Bidirectional scan mode (16 Bit) was used with 1X averaging applied. Raw Z-stacks were then processed using Zen 3.4 (blue edition) to apply 3D super resolution post-processing. These images were then analysed using Bitplane Imaris software to generate maximum intensity projections. The electronically switchable illumination and detection module (ESID) was also used to collect transmitted light (**Figure S1**).

7. Read the plate immediately with the microplate reader.

### Spectrofluorometer setup and operation

1. Fluorescence kinetic readings were collected over time with multiple readings during 6 h with intervals of 4 min at maximal readings per well at 37°C (**Figure 1.7**).

2. Multiple-wavelength readings (excitation/emission) were performed at the same time. Optimal measurements were taken using the desired wavelengths as follows:

· Ex/Em 585/625 nm for DQ red BSA

· Ex/Em 395/435 nm for AF405

· Ex/Em 490/530 nm for pHrodo green

Note. Not shake the plate. The plate should be read from the bottom with a photomultiplier tube (PMT) in low fluorescence gain to avoid signal saturation.

### Assessment of phagosomal pH

**Figure 2:**
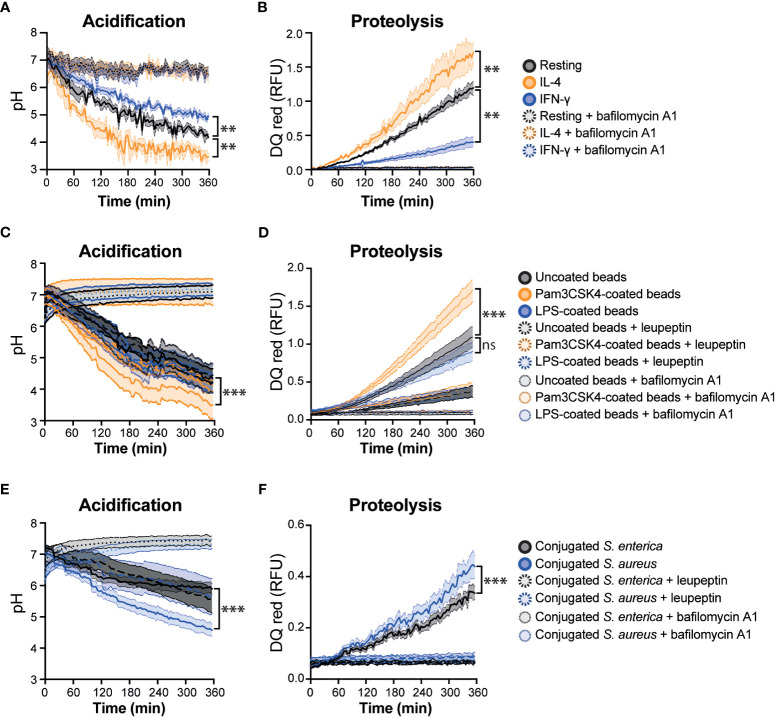
Real-time multiplex phagosome acidification and proteolysis analysis in RAW 264.7 cells. **(A)** Acidification and **(B)** proteolysis were measured in RAW 264.7 cells untreated (resting) or treated with 20 μg/mL IFN-γ or 20 μg/mL IL-4 for 24 hours. **(C)** Acidification and **(D)** proteolysis were measured in untreated RAW 264.7 cells with uncoated, Pam3CSK4-coated or LPS-coated bi-functional beads. **(E)** Acidification and **(F)** proteolysis were measured in untreated RAW 264.7 cells with conjugated-*Salmonella enterica* (*S. enterica*) or *Staphylococcus aureus* (*S. aureus*). Bafilomycin A1 and leupeptin were used as a negative control of phagosome acidification and proteolysis, respectively, which are two aspects of phagosome maturation, but it does not completely block the overall process of phagosome maturation. Friedman one-way ANOVA test followed by Dunn *post hoc* test. The statistical significance of the comparisons with resting is indicated as follows: **P ≤ 0.01; ***P ≤ 0.001; ns, not significant. Error bars represent SEM of six biological replicates. RFU, relative fluorescence units.

The average of three technical replicates were calculated and blank values were subtracted from each data set. The relative fluorescent units (RFUs) were calculated by normalising pHrodo green dye intensity against the calibration Alexa Fluor 405 intensity. To calculate the pH, a cubic polynomial regression curve (*f p(x): ax^3^ + bx^2^ + cx + d*) was analysed by obtaining measurements of the coupled particles at a known pH range (7.5 – 4.0) in each experiment to assess precision. The RFUs from six biological replicates were then interpolated to this pH curve to obtain the acidification results (**Figures 2A, C, E**, **Figures S2A, C, E**). Excitation/emission corresponding to DQ red BSA (585/625 nm) was not affected by pH (**Figure S3A**).

### Assessment of phagosomal proteolysis

To measure the phagosomal proteolysis the same approach for pHrodo green was followed with DQ red BSA. In short, blank values were subtracted from each data set. The RFUs from six biological replicates were calculated by normalising DQ red BSA dye intensity against the calibration Alexa Fluor 405 intensity (**Figures 2B, D, F**, **Figures S2B, D, F**). To validate the proteolysis of DQ red BSA, the particles were incubated at 37°C with 1 μg/μL trypsin TPCK (Worthington-Biochem) for 2 h to induce the proteolysis of BSA and measure in the spectrofluorometer. Excitation/emission corresponding to pHrodo Green (490/530 nm) was not affected by trypsin (**Figure S3B**).

### Flow cytometry

For flow cytometry analysis, the cells were washed twice with warm PBS, trypsinised, and analysed in a FACSymphony A5 flow cytometer (Becton-Dickinson) using the high-throughput sampler (HTS) unit. Cells were selected based on SSC-A/FSC-A properties. Singlets were gated as FSC-H/FSC-A, then the excitation-emission (Ex/Em) 480/530 nm for pHrodo green, Ex/Em 635/670 nm for DQ red BSA, and Ex/Em 405/450 nm for AF405 were measured. The results were analysed using FlowJo V10 (**Figure S4**).

### Statistical analysis

Statistical analyses were performed in GraphPad Prism (version 9.3.1). A nonparametric test was used for paired data: Friedman one-way ANOVA test followed by Dunn *post hoc* test; p-value < 0.05 was considered statistically significant.

## Discussion

We described in detail and developed a method to prepare a bi-functional particle probe to simultaneously measure acidification and proteolysis during phagosome maturation in macrophages. This assay measures in real time the gradual reduction in pH during phagosomal acidification and increase in proteolysis, respectively. Generally, compared to existing techniques for measuring chemical reactions within living cells, the pH of the phagosome is measured with pH-sensitive pHrodo dye or carboxyfluorescein, attached to microscopic beads, which will emit higher fluorescence when exposed at acidic pHs (13–15, 18, 21). However, pHrodo-labelled bioparticles showed a more precise and efficient quantification of phagosomal pH, under different assays, such as spectrofluorometry and flow cytometry (22). Our assay combines multiple fluorescent dyes to measure the pH and protein content inside the phagosome in real-time. For proteolysis, DQ red BSA was used. The close proximity of the dye molecules on BSA quenches the fluorescence while BSA is intact and when digested by acidic hydrolases found in the phagolysosome, its fluorescence increases when DQ red containing peptides are released (14). This combination of fluorescent dyes provides a powerful tool for the simultaneous monitoring of two important processes in a single sample. However, recent studies (17, 23–25) have emphasized single-cell analysis because phagosomes within cells can exhibit heterogeneity in their maturation processes. Averaged measurements can mask important differences between individual phagosomes and their respective cellular contexts. By studying single phagosomes at the single-cell level, researchers can capture the diversity of phagosome maturation dynamics and understand the heterogeneity within a population of cells. To address this, our method is compatible with flow cytometry and fluorescence microscopy, allowing us to quantify phagosome maturation in single cells and visualise the dynamics of phagocytosis over time, respectively. Consistent with existing literature, our observations demonstrated that M2 macrophages showed higher phagosome maturation capacities compared to M1 macrophages (26). This enhanced maturation was characterised by increased lysosome fusion and acidification within the phagosomes of M2 macrophages. Moreover, phagosome maturation increases with Pam3CSK4-coated beads as it has been identified previously (27, 28). By employing different cellular models, we aimed to evaluate the assay’s robustness, reproducibility, and potential variations across different cell types. This approach helps in establishing the assay’s general applicability and understanding its behaviour in various biological contexts. In addition, this method can be used to assess the efficacy of drugs or therapeutic compounds in promoting or inhibiting phagosome acidification and proteolysis. This information can help identify potential drug candidates for modulating immune responses or treating infectious diseases.

The assay is able to accurately assess changes in both phagosome acidification and proteolysis and can be adapted for use in a variety of cell types. Moreover, the method’s compatibility with flow cytometry and adaptability to high-throughput experiments make it suitable for large-scale screening of compounds or genetic factors that impact phagosome acidification and proteolysis. This new approach to multiplexing will enable researchers to assess the effects of external stimuli rapidly and accurately on phagosome acidification and proteolysis in a wide range of cells and organisms. Lastly, because this method can be applied to labelled bacteria or apoptotic/necroptotic cells, this technique will be broadly applicable to determine the mechanisms of action of pathogen cells that evade host immunity by manipulating phagosome functions.

## Data availability statement

The raw data supporting the conclusions of this article will be made available by the authors, without undue reservation.

## Ethics statement

The animal study was reviewed and approved by NewcastleUniversity.

## Author contributions

AM-A and JM-R performed and analysed most experiments. BR performed additional experiments. JM-R, AM-A, and MT wrote the manuscript. MT and JM-R conceived the original idea and provided supervision. All authors provided critical feedback and helped shape the research, analysis, and manuscript.
