# Supplementary material for: Bi-functional particles for real-time phagosome acidification and proteolysis multiplex assay in macrophages
Source: Front Immunol. 2023 Aug 10;14:1204223. doi: 10.3389/fimmu.2023.1204223 (PMC10456865; doi:10.3389/fimmu.2023.1204223)
Supplement: Supplementary file 1 [file DataSheet_1.pdf]

Supporting Information

Bi-functional particles for real-time phagosome acidification and proteolysis multiplex assay in macrophages

Alba Méndez-Alejandre[1,2,#], Benjamin Bernard Armando Raymond[1], Matthias Trost[1,*], José Luis Marín-Rubio[1,*,#]

[1] Laboratory for Biological Mass Spectrometry, Biosciences Institute, Newcastle University, Newcastle-upon-Tyne, NE2 4HH, UK.

[2] Biology Department, Autonomous University of Madrid, Madrid, 28049, Spain.

#These authors contributed equally to this work.

* Corresponding authors:

José Luis Marín-Rubio. E-mail: jose.marin-rubio@newcastle.ac.uk

Matthias Trost. E-mail: matthias.trost@ncl.ac.uk

CONTENT:

Figure S1. Bi-functional particles in cells.

Figure S2. Real-time multiplex phagosome acidification and proteolysis analysis in BMDMs and in the human non-adherent monocytic cell line, THP-1.

Figure S3. Quality control of bi-functional particles.

Figure S4. Phagosome acidification and proteolysis analysis by flow cytometry.

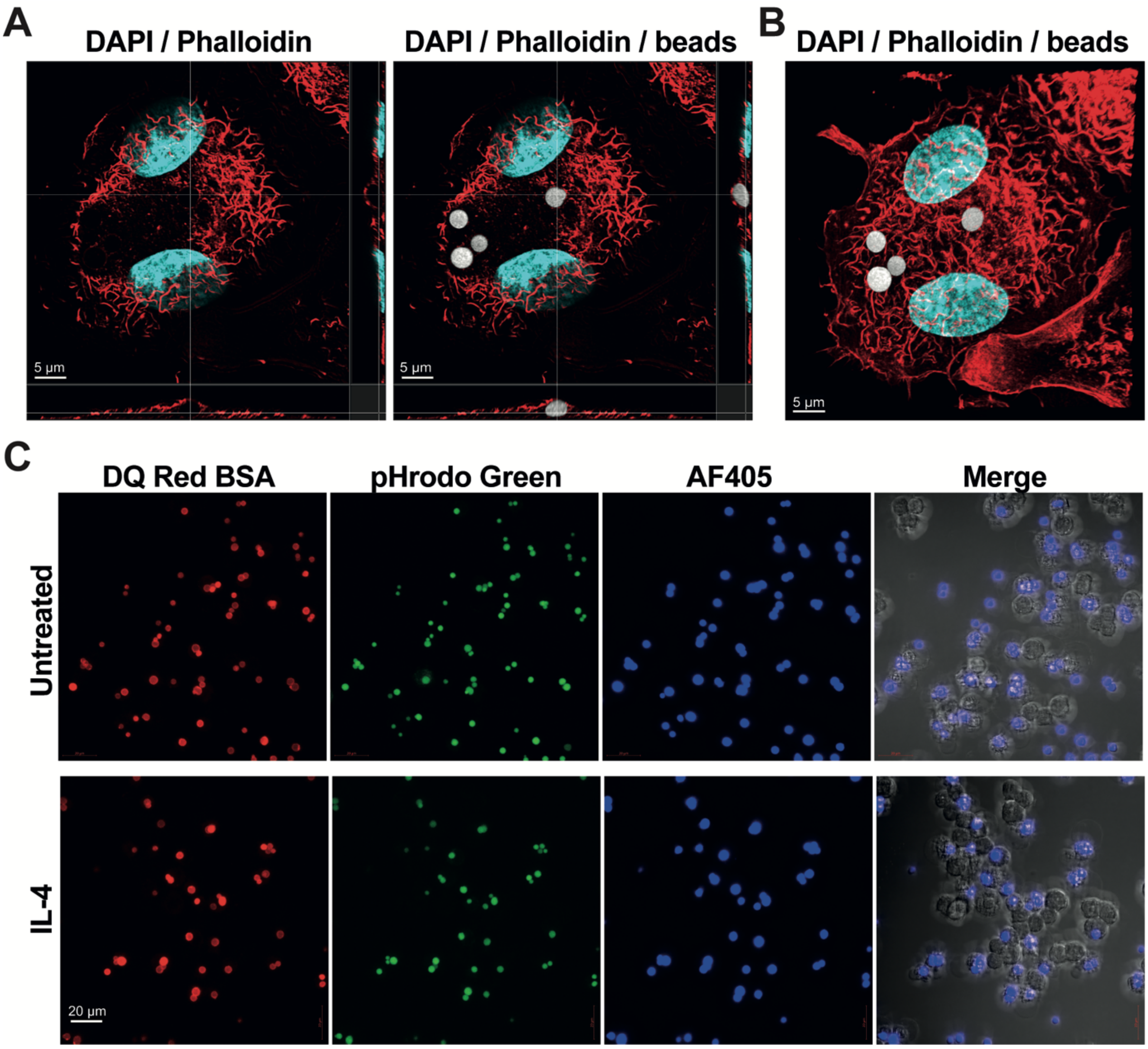

Figure S1. Bi-functional particles in cells. A) Confocal xyz slice view of THP-1 cells after 30 min uptake of bi-functional particles (white). **B)** Maximum intensity projection of a cell with beads (white). DAPI in turquoise and phalloidin in red. Scale bar represents 5 µm. Representative images are shown. **C)** Immunofluorescence microscopy was performed in RAW 264.7 cells after 6 h post-uptake of bi-functional particles in untreated (resting) or treated with 20 µg/mL IFN-γ. DAPI in blue, pHrodo green in green, DQ red BSA in red, electronically switchable illumination and detection image (ESID) in white. Representative images are shown. Scale bar represents 20 µm.

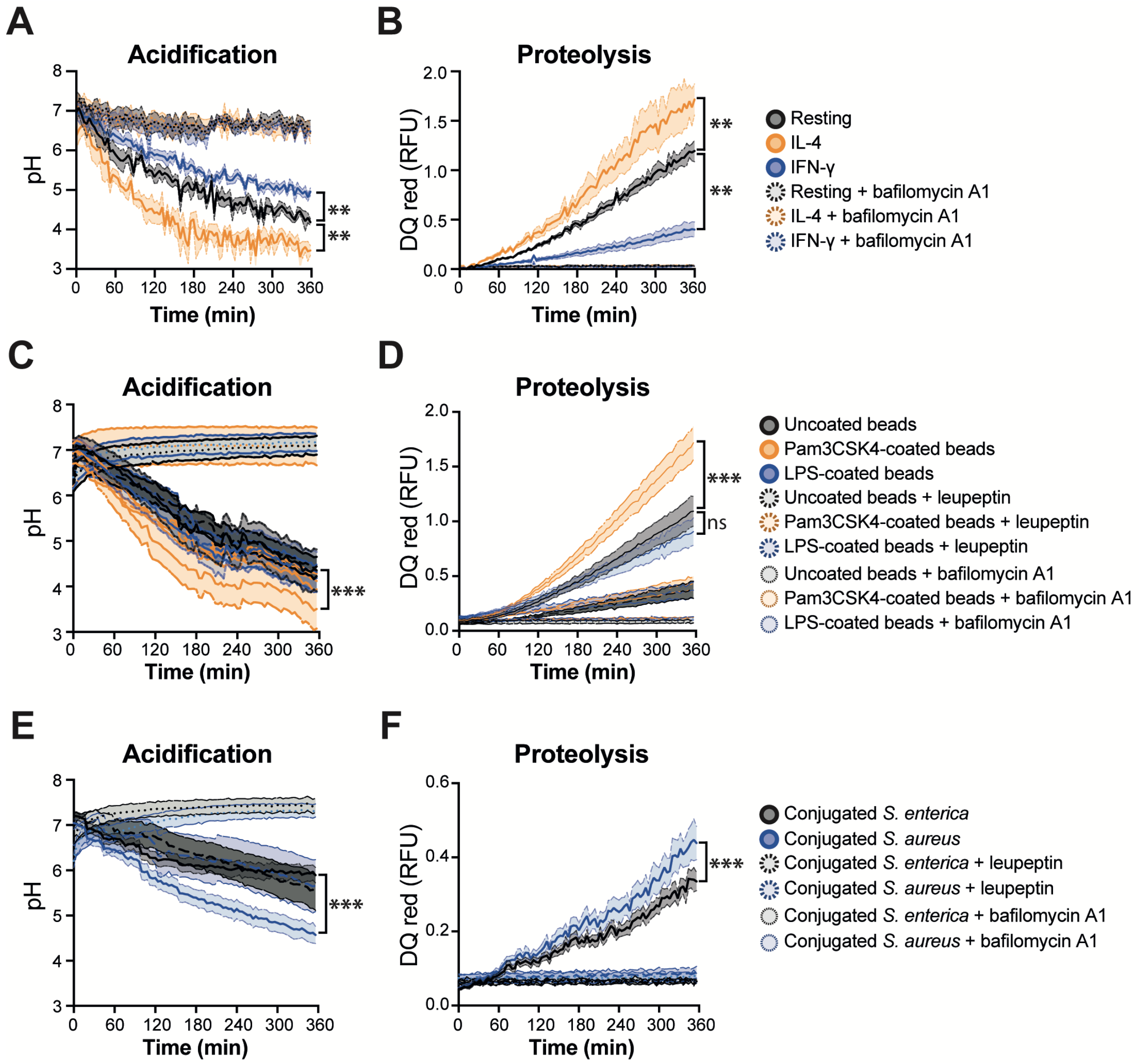

Figure S2. Real-time multiplex phagosome acidification and proteolysis analysis in RAW 264.7 cells and in the human non-adherent monocytic cell line, THP-1. A-D) Acidification and proteolysis were measured in BMDMs untreated (resting) or treated with 20 µg/mL IFN-γ or 20 µg/mL IL-4 for 24 h (**A-B**) and 48 h (**C-D**). **E)** Acidification and **F)** proteolysis were measured in untreated THP-1 cells with uncoated, Pam3CSK4-coated or LPS-coated bi-functional beads. Bafilomycin A1 and leupeptin were used as a negative control of phagosome acidification and proteolysis, respectively, which are two aspects of phagosome maturation, but it does not completely block the overall process of phagosome maturation. Friedman one-way ANOVA test followed by Dunn post hoc test. The statistical significance of the comparisons with resting is indicated as follows: *, $P \leq 0.05$; **, $P \leq 0.01$; ***, $P \leq 0.001$; ns, not significant. Error bars represent SEM of six biological replicates.

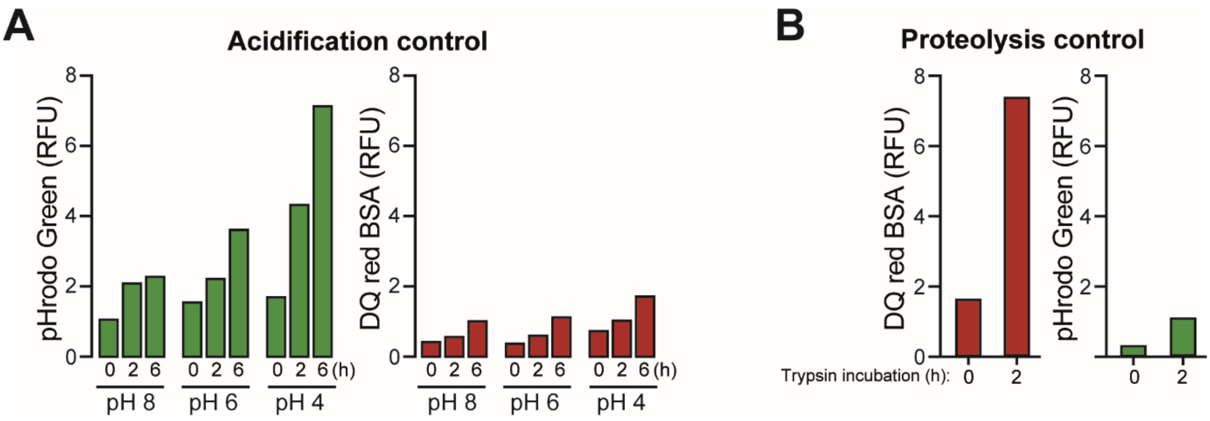

Figure S3. Quality control of bi-functional particles. A) Differet pH buffers for 6 h increased relative fluorescence units (RFU) in pHrodo green (490/530 nm) but not red fluorescence (585/625 nm). **B)** Bi-functional particles incubated for 2 h at 37°C with 1 µg/µL trypsin increased DQ red BSA fluorescence (585/625 nm), but not green fluorescence (490/530 nm).

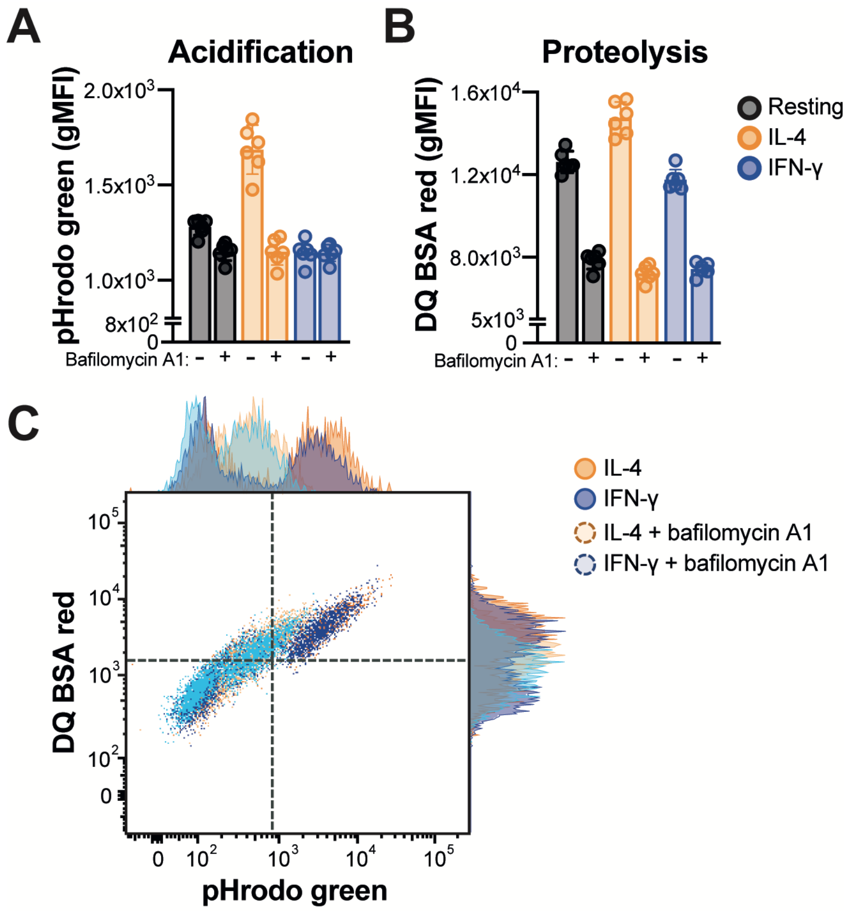

Figure S4. Phagosome acidification and proteolysis analysis by flow cytometry. **A)** Acidification and **B)** proteolysis were measured in BMDMs untreated (resting) or treated with 20 µg/mL IFN-γ or 20 µg/mL IL-4 for 48 h. Error bars represent SEM of six biological replicates. **C)** Representative dot-plot and histogram charts for DQ BSA red and pHrodo green. Bafilomycin A1 was used as a negative control of phagosome acidification, which is one aspects of phagosome maturation, but it does not completely block the overall process of phagosome maturation.
